# Effects of Dietary Cholesterol and Its Oxidation Products on Pathological Lesions and Cholesterol and Lipid Oxidation in the Rabbit Liver

**DOI:** 10.1155/2014/598612

**Published:** 2014-02-20

**Authors:** Sun Jin Hur, Ki Chang Nam, Byungrok Min, Min Du, Kwon Il Seo, Dong Uk Ahn

**Affiliations:** ^1^Department of Animal Science and Technology, Chung-Ang University, 4726 Seodong-daero, Daedeok-myeon, Anseong-si, Gyeonggi-do 456-756, Republic of Korea; ^2^Department of Animal Science and Technology, Sunchon National University, Suncheon 540-742, Republic of Korea; ^3^Food Science and Technology Program, Department of Agriculture, Food, and Resource Sciences, University of Maryland Eastern Shore, Princess Anne, MD 21853, USA; ^4^Department of Animal Science, Washington State University, Pullman, WA 99164-6310, USA; ^5^Department of Food and Nutrition, Sunchon National University, Suncheon 540-742, Republic of Korea; ^6^Department of Animal Science, College of Agriculture, Iowa State University, Ames, IA 50011, USA

## Abstract

This study was conducted to determine the effects of dietary cholesterol (CHO) and cholesterol oxidation products (COPs) on the induction of pathological lesions in rabbit liver tissues. Liver lesions were induced only when the levels of CHO and COPs in the diet were very high. The amount of CHO measured in the liver increased when dietary CHO was increased; by comparison, dietary COPs affected liver CHO amounts to a lesser extent. The TBARS (thiobarbituric acid reactive substances) value measured for the liver samples also increased when dietary CHO and COP levels were elevated, and the TBARS value was more strongly affected by the amount of COPs in the diet than by the amount of CHO. At 6 and 12 weeks, COP levels were the highest in the group that received 1.2 g CHO + 0.8 g COPs, followed by the 0.5 g CHO + 0.5 g COPs and 1.6 g CHO + 0.4 g COPs groups; the control (0 g) group showed the lowest COP levels among all groups. In this study, we found that not only dietary CHO but also COPs were involved in hypercholesterolemia induced liver lesions when the amount of CHO and COPs was high.

## 1. Introduction

Cholesterol (CHO) is a crucial component of the human body, but a high level of CHO is considered a major risk factor for the development of atherosclerosis and coronary heart disease (CHD) [1]. Moreover, the intake of cholesterol oxidation products (COPs) also typically leads to the development of atherosclerosis and CHD [2]. CHO is widely distributed in living organisms and is localized mainly in cell membranes in its nonesterified form [3] and in the blood as a component of lipoproteins mainly in its esterified form [4], and CHO plays a role in numerous physiological and pathological processes. CHO may be derived either from the diet or by means of endogenous synthesis, which occurs primarily in the liver [4]. The liver is also the major site where CHO is removed by being directly secreted into the bile or by being broken down into bile acids; moreover, the liver plays a key role in maintaining whole body CHO homeostasis by controlling the uptake of extracellular CHO, CHO synthesis, and CHO storage [5]. An elevated level of plasma CHO is typically a risk factor for atherosclerosis, and several COPs are cytotoxic, atherogenic, mutagenic, or carcinogenic; furthermore, COPs injure endothelial cells, leading to atherosclerosis [1]. Alterations in hepatic CHO homeostasis caused by dietary or drug interventions potently influence CHO balance and plasma low density lipoprotein (LDL) CHO levels in the body [6]. Thus, dietary CHO and COPs critically affect health because LDL CHO levels are positively correlated with the risk of cardiovascular disease. Metabolic changes in liver tissues serve as a barometer of the potential risk of atherosclerosis and CHD, but the relationship between atherosclerosis and metabolic changes in liver tissues, CHO type, and changes in liver lipids is not well understood. In this study, our objective was to determine the relationship between dietary CHO and COPs and the induction of lesions in liver tissues and the composition of CHO, COPs, TBARS, and fatty acids in the liver tissues of rabbits.

## 2. Materials and Methods

### 2.1. Animal Diets and Experimental Protocol

We divided 64 young male New Zealand White rabbits (average weight 3 kg) into 8 groups. Each group was balanced with respect to bodyweight by means of a restricted randomization technique, and the rabbits were housed individually in stainless steel cages during the 3-month feeding trial. After 1 week of acclimation, each group of rabbits was fed a commercial rabbit chow containing no additives (0 g CHO + 0 g COPs) or chow containing, per kg diet, 1 g CHO, 2 g CHO, 0.9 g CHO + 0.1 g COPs, 0.8 g CHO + 0.2 g COPs, 0.5 g CHO + 0.5 g COPs, 1.6 g CHO + 0.4 g COPs, or 1.2 g CHO + 0.8 g COPs. Diets were prepared at 2-week intervals and stored in a cold room (0.5°C) after vacuum packaging (1 bag/day). Four rabbits per treatment were sacrificed by means of pentobarbital overdose (200 mg/kg bodyweight) after 45 and 90 days of feeding. After liver sampling, the thorax was opened and liver tissues were collected. Feeding, sample collection, and euthanasia protocols were all approved by the Animal Care Committee of Iowa State University and complied with the Care and the Use of Laboratory Animals. The nutrient content of the basal diet used in this study is listed in Table 6.

### 2.2. Preparation of Dietary COPs

COPs were prepared by heating CHO with H_2_O_2_ at 150°C for 72 h. As evaluated using gas chromatography (GC 6890, Agilent, Wilmington, DE, USA) mass spectrometry, the final extract contained 7*α*-hydroxycholesterol (8.5%), 7*β*-hydroxycholesterol (14.8%), 5*α*- and 6*α*-epoxycholesterol (18.8%), 5*β*- and 6*β*-epoxycholesterol (20.2%), a mixture of 6-ketocholesterol and 25-hydroxycholesterol (6.3%), and 7-ketocholesterol (24.8%).

### 2.3. Pathological Liver Lesions

Light microscopy was used to determine the pathologic conditions and the degree of lesions in liver tissues that were caused by the dietary treatments. Liver tissues were fixed in 10% buffered formalin for 24 h, washed for 4 h in tap water, dehydrated in an automated processor, and embedded in paraffin. Next, 4 *μ*m thick sections were cut and stained with hematoxylin and eosin (HE), periodic acid-Schiff (PAS)-Alcian blue at pH 2.5, and oil red O to observe the pathological lesions present in the liver tissues.

### 2.4. Liver Cholesterol Analysis

We added 2 g of liver samples to a 50 mL tube containing 10 mL of a saponification reagent (30% KOH and ethanol at 6 : 94 ratio) and 0.5 mL of an internal standard (2 mg of 5*α*-cholestane/sample) and homogenized the samples for 10 s using a Polytron homogenizer (IKA Labortechnik T25-B, Staufen im Breisgau, Germany); the tubes were then capped and the samples were incubated for 1 h at 60°C. After cooling, 8 mL of deionized distilled water and 3 mL of hexane were added and the samples were mixed thoroughly and allowed to separate. The top layer (hexane layer) was collected and dried in scintillation vials. Next, 100 *μ*L of bis-[trimethylsilyl]trifluoroacetamide (BSTFA) + 1% trimethylchlorosilane (TMCS) and 200 *μ*L of pyridine were added, and the samples were mixed and incubated overnight and then were analyzed using gas chromatography (GC 6890, Agilent). A ramped oven temperature condition was used (180°C for 2.5 min, increased to 230°C at 2.5°C/min, and then held at 230°C for 7.5 min), and the temperature of both the inlet and the detector was 280°C. Helium was used as the carrier gas at a linear flow of 1.1 mL/min; the detector-air (flame ionization detector, FID), H_2_, and make-up gas (He) flows were 350, 35, and 43 mL/min, respectively.

### 2.5. Analysis of TBARS in Liver

A modified fluorometric method [7] was used for tissue TBARS analysis. Each sample (0.5 mL of tissue homogenate in 4 vol. of water) was transferred into a 15-mL tube and mixed with 200 mL of 8.1% sodium dodecyl sulfate, 1.5 mL of HCl, 1.5 mL of 20 mM thiobarbituric acid, 50 mL of butylated hydroxytoluene (BHT; 10% in 95% ethanol), and 250 mL of double distilled water (DDW). Samples were vortexed and then heated in a 90°C water bath for 15 min. After cooling for 10 min, 1 mL of DDW and 5 mL of n-butanol/pyridine solution were added, and the samples were mixed thoroughly and centrifuged at 3,000 ×g for 15 min. The fluorescence reading of the upper layer was measured using a fluorometer at an excitation wavelength of 520 nm and emission wavelength of 550 nm. TBARS levels in liver tissues were expressed as mg malondialdehyde (MDA)/kg tissues.

### 2.6. Analysis of COPs in Liver

The COPs present in the liver were measured using a method described by Ahn et al. [8]. Extracted lipids [9] were dissolved in hexane at a final concentration of 0.1 g fat/mL hexane and used in the following step. A mixture of silicic acid (100 mesh), celite 545, and CaHPO_4_ (10 : 9 : 1, w/w/w) was prepared in chloroform and packed into glass columns. The columns were washed with 10 mL of hexane  : ethyl acetate (9 : 1 v/v, Solvent 1) before loading samples. Lipid samples (0.2 g) dissolved in hexane were mixed with an internal standard (5-*α*-cholestane) and loaded onto the silicic acid columns. Neutral lipids, CHO, and phospholipids were eluted by passing hexane  : ethyl acetate (9 : 1, v/v) and hexane  : ethyl acetate  : diethyl ether (4 : 1 : 2, v/v/v) through the column. Next, COPs were eluted using acetone: ethyl acetate  : methanol (10 : 10 : 1) and dried under nitrogen. Dried COPs were added to 200 *μ*L of pyridine and 100 *μ*L of BSTFA + 1% TMCS and derivatized by heating in a dry bath (80°C) for 1 h and analyzed using a gas chromatography system (GC 6890, Agilent) equipped with an on-column capillary injector and an FID. A ramped oven temperature was used (180°C for 2.5 min, increased to 230°C at 2.5°C/min, and then held at 230°C for 7.5 min), and the temperature of both the inlet and the detector was 280°C. Helium was used as the carrier gas at a linear flow of 1.1 mL/min. The detector-air (FID), H_2_, and make-up gas (He) flows were 350, 35, and 43 mL/min, respectively. Fatty acids were identified by comparing their retention times with those of established standards, and their relative quantities were expressed as the weight percent of total fatty acids.

### 2.7. Analysis of Liver Fatty Acids

The extracted lipids were dissolved in hexane to obtain a final concentration of 0.1 g fat/mL hexane and were then used in the following step. We added 1 mL of hexane and 1 mL of a methylating reagent (3N-HCl in methanol) to 100 *μ*L of lipid extracts (of liver) and incubated the samples at 90°C in a water bath for 1 h. After cooling to room temperature, 2 mL of hexane and 5 mL of water were added to the samples, which were mixed thoroughly and incubated at room temperature overnight to allow phase separation. The top hexane layer containing the methylated fatty acids was analyzed for fatty acid composition by using a gas chromatograph (GC 6890, Agilent). Gas chromatography conditions were the same as those used for COPs analysis.

### 2.8. Statistical Analysis

Data were analyzed using SAS software [10] by using the generalized linear model procedure. The Student-Newman-Keuls multiple range tests were used to compare differences among means. Mean values and standard deviation of the mean (STD) are reported. Statistical significance was defined as *P* < 0.05.

## 3. Results and Discussion

### 3.1. Pathological Lesions in Liver

The histopathological results obtained are summarized in Table 1. The histopathological data (qualitative) indicated that the rabbits in the groups that received 1.6 g CHO + 0.4 g COPs and 1.2 g CHO + 0.8 g COPs exhibited the most severe vacuolation of hepatocytes in the periacinar region of the hepatic lobule, followed by the 2 g CHO group; however, the 0-g group showed no abnormalities at 6 or 12 weeks. The rabbits that were fed with the diets containing (per kg diet) 1 g CHO, 0.9 g CHO + 0.1 g COPs, 0.8 g CHO + 0.2 g COPs, and 0.5 g CHO + 0.5 g COPs showed the least vacuolation of hepatocytes in the periacinar region of the hepatic lobule at 6 and 12 weeks. After 12 weeks, rabbits of the groups fed with 1.6 g CHO + 0.4 g COPs and 1.2 g CHO + 0.8 g COPs developed diffuse severe vacuolation of hepatocytes throughout all areas of the hepatic lobule and exhibited marked hepatocyte swelling.

### 3.2. Cholesterol Content in Liver

The liver CHO content measured at 6 weeks and at the end of the experiment is presented in Table 2. CHO content increased significantly (*P* < 0.05) with an increase in dietary CHO and COPs in all diet groups, but the CHO content did not change significantly between feeding periods in the group that received 1.2 g CHO + 0.8 g COPs. At both 6 and 12 weeks, high levels of CHO in the diet significantly increased (*P* < 0.05) CHO content in the liver compared with low levels of CHO in the diet. The group that received 2 g CHO diet showed a significantly higher (*P* < 0.05) CHO content in the liver than did all the other groups, because this diet contained the highest amount of CHO among all the diets tested.

### 3.3. TBARS Contents in Liver

Table 3 shows the effect of dietary CHO and COPs on TBARS values, which were used as a measure of lipid oxidation in the liver. The TBARS values in the liver increased significantly (*P* < 0.05) with an increase in dietary intake of CHO and COPs. TBARS values have been shown to increase with lengthening of feeding periods. At 6 weeks, TBARS levels in the liver were significantly higher (*P* < 0.05) in the 1.2 g CHO + 0.8 g COPs diet group than in the other diet groups. At 12 weeks, the 1.2 g CHO + 0.4 g COPs diet group showed the highest levels of TBARS (*P* < 0.05), followed by the 1.6 g CHO + 0.4 g COPs, 0.5 g CHO + 0.5 g COPs, 2 g CHO, and 0.8 g CHO + 0.2 g COPs diet groups, and then the remaining diet groups.

### 3.4. Cholesterol Oxide Product Contents in Liver

Table 4 shows the effect of dietary CHO and COPs on COP content in the liver. COP levels increased significantly (*P* < 0.05) with an increase in feeding periods in all diet groups. Both at 6 and 12 weeks, COP levels were the highest (*P* < 0.05) in the 1.2 g CHO + 0.8 g COPs group, followed by the 0.5 g CHO + 0.5 g COPs group or 1.6 g CHO + 0.4 g COPs group; the 0 g group showed the lowest COP levels (*P* < 0.05) among all diet groups.

### 3.5. Fatty Acid Compositions in Liver

The composition of fatty acids in the liver was markedly influenced by dietary CHO and COPs (Table 5). Unsaturated fatty acids increased substantially, whereas saturated fatty acids decreased considerably. Dietary COPs more strongly affected the fatty acid composition in the liver than CHO did. The percentage of palmitoleic acid and linolenic acid increased (*P* < 0.05), whereas that of stearic acid decreased (*P* < 0.05) with an increase in dietary CHO and COPs.

Hypercholesterolemia and hyperlipidemia caused by dietary CHO and COP intake can potently influence lipid metabolism in the liver. Gordon et al. [11] reported that CHO-fed New Zealand White rabbits displayed distortions in hepatic cellular architecture that resulted from severe fat deposition in the liver. Subramanian et al. [12] also determined that dietary CHO induced a high degree of inflammation, fibrosis, and hepatocyte injury in the mouse liver. In this study, dietary CHO and COPs modified liver lipid metabolism and induced liver lesions in rabbits; both COPs and CHO induced pathological lesions in the liver when their dietary levels were high (the total amount of CHO and COPs was 2 g). Specifically, the amounts of COPs in the diet exhibited a larger effect than CHO did on increasing pathological lesions in the liver. Subramanian et al. [12] suggested that the COPs generated by means of autooxidation or enzymatic/nonenzymatic peroxidation of sterols also trigger apoptosis and liver injury. We surmise that dietary CHO and COPs, influenced largely by oxidative stress, affect the induction of liver lesions. In this study, dietary CHO and COPs increased lipid-oxidation values (TBARS) in the liver; the amount of COPs in the diet exerted a stronger effect than CHO did on increasing the TBARS values. Hurst et al. [13] reported that cellular damage can be produced by various activated species including superoxide, hydrogen peroxide, peroxynitrite, and singlet oxygen. The peroxidation of membrane lipids and the accumulation of lipid oxidation products such as MDA and oxysterols generally induce spontaneous oxidation of cellular cholesterol and trigger cell death [14]. Therefore, liver lesions were expected to be elevated when TBARS values were increased as a result of including COPs in the diet in this study.

COPs are more injurious to arterial cells than is pure CHO, and COPs are more directly linked than CHO is to the development of atherosclerosis and coronary heart disease [15]. COPs also impair membrane function, which results in altered membrane permeability [16]. Smith [17] reported that natural CHO exerted no atherogenic or hypercholesterolemic effect and that CHO influenced the activities of most enzymes to a lesser extent than COPs did. By contrast, Kleemann et al. [18] reported that nutritional CHO itself may contribute to the evolution of the inflammatory component of atherogenesis, and that proatherogenic inflammatory factors originated at least in part from the liver. Vejux and Lizard [14] also reported that oxysterols potently induce apoptosis, and that they can also induce necrosis in various cell types, with this mode of cell death potentially being more pronounced at high oxysterol concentrations. Our previous study [1] showed that both dietary COPs and high CHO levels are associated with an increased risk of atherosclerosis in rabbits, and this study has revealed that not only dietary COPs but also CHO induced pathological lesions in the liver. In this study, hypercholesterolemia induced liver lesions when the levels of dietary CHO and COPs were high (the total amount of CHO and COPs was 2 g). Kleemann et al. [18] also demonstrated that high-dose dietary CHO strongly induces early atherosclerotic lesion formation in a mouse model. These results indicate that the risks of liver lesions and atherosclerotic lesions imposed by dietary CHO and COPs are closely related and that hepatic lesions may be causatively related to atherosclerosis.

Aghaei et al. [19] reported that dietary CHO markedly altered fatty acid compositions in animal liver. This study revealed that dietary CHO and COPs increased unsaturated fatty acids and decreased saturated fatty acids. The percentage of palmitoleic acid and linolenic acid increased (*P* < 0.05), whereas that of stearic acid and arachidonic acid decreased (*P* < 0.05) with an increase in dietary CHO and COPs. The reason why unsaturated fatty acids are increased by dietary CHO and COPs remains unclear, but the changes in lipid composition induced by dietary CHO may be caused by compensatory mechanisms initiated to maintain lipid homeostasis in cellular aspects such as membrane fluidity [20].

Unsaturated fatty acids are molecules that are typically unsaturated or contain double bonds and are therefore prone to oxidation [1]. Moreover, unsaturated fatty acids are sensitive to free-radical oxidation caused by diatomic molecular oxygen, and oxidized fatty acids play a major role in the action of oxidized LDL [1]. Rudel et al. [21] reported that LDL particles enriched in polyunsaturated fatty acids are also easily oxidized in vitro. Therefore, dietary COPs and CHO may influence the oxidation of unsaturated fatty acids and thereby lead to an increase in liver lesions. In this study, dietary COPs, compared with dietary CHO, more strongly influenced fatty acid composition in the liver. Thus, oxidized CHO may affect fatty acid oxidation and the formation of liver lesions. Staprans et al. [22] reported that oxidized CHO is absorbed and that it contributes to the pool of oxidized lipids in circulating lipoproteins. When rabbits were fed with oxidized CHO, fatty streak lesions in the aorta were increased by 100% [22]. Staprans and coworkers hypothesized that both diet-derived oxidized fatty acids in chylomicron remnants and oxidized cholesterol in the remnants and in LDL accelerate atherosclerosis by increasing oxidized lipid levels in circulating LDL and chylomicron remnants. In this regard, we surmise that increases in unsaturated fatty acid levels are influenced largely by dietary COPs rather than CHO, and that dietary COPs contribute to the oxidation of CHO and liver lesions in rabbits. This is another possible mechanism by which dietary COPs and high natural CHO levels are associated with the increased risk of liver lesions in rabbits. However, changes in fatty acid composition are not influenced by only a single factor. Thus, additional studies are required to understand the relationship between dietary CHO or COPs and fatty acid composition in the liver.

## 4. Conclusion

CHO levels in the liver were significantly increased when the amounts of dietary CHO were increased; by comparison, increasing dietary COPs affected liver CHO levels to a lesser extent. TBARS and COP levels in the liver were elevated with increases in dietary CHO and COPs, and the amount of COPs in diet exerted a larger effect than CHO did on increasing the contents of TBARS and COPs in the liver. The percentages of palmitoleic acid and linolenic acid increased, whereas that of stearic acid decreased with increasing dietary CHO and COPs. In this study, we determined that not only dietary CHO but also COPs were involved in hypercholesterolemia-induced liver lesions when the amount of CHO and COPs was high. However, dietary COPs exhibited a greater influence than did CHO on liver cell damage.

## Figures and Tables

**Table 1:** Effect of dietary cholesterol and cholesterol oxidation products on liver lesions: pathological lesions in liver.

Dietary		
CHO1 + COPs g/kg diet	6 weeks	12 weeks
0 g	0 0 0 0	0 0 0 0

1 g chol.	+ + + +	+ + + +

2 g chol.	+ ++ ++ ++++	++ ++ ++ +++

0.9 chol. + 0.1 COPs	+ + + +	+ + + +

0.8 chol. + 0.2 COPs	+ + + +	+ + + +

0.5 chol. + 0.5 COPs	+ + + +	+ + + +

1.6 chol. + 0.4 COPs	+ ++ +++ ++++	++ +++ +++ ++++

1.2 chol. + 0.8 COPs	+ +++ +++ ++++	++ +++ ++++ ++++

0: no abnormalities detected; +: mild vacuolation of hepatocytes in the periacinar region of the hepatic lobule; ++: mild vacuolation of hepatocytes in the periacinar and midzonal areas of the hepatic lobule; +++: moderate vacuolation of cells in the periacinar and midzonal areas of the hepatic lobule, with affected cells showing diffuse vacuolation of the cytoplasm; ++++: diffuse and severe vacuolation of hepatocytes in all areas of the hepatic lobule, with marked swelling of the hepatocytes.

^1^CHO: natural cholesterol; COPs: cholesterol oxidation products.

**Table 2:** Effect of dietary cholesterol and cholesterol oxidation products on cholesterol content in liver.

	Cholesterol in liver (mg/g)		
Dietary			
CHO^1^ + COPs g/kg diet	6 weeks	12 weeks	SEM
0 g	1.123^by^	3.120^az^	0.156
1 g	3.231^bx^	16.727^aw^	0.405
2 g	17.795^bv^	27.735^av^	0.940
0.9 chol. + 0.1 COPs	2.787^bx^	10.737^ax^	0.813
0.8 chol. + 0.2 COPs	2.271^bxy^	9.004^axy^	0.714
0.5 chol. + 0.5 COPs	2.580^bx^	6.890^ay^	0.948
1.6 chol. + 0.4 COPs	11.072^bw^	16.218^aw^	0.894
1.2 chol. + 0.8 COPs	10.558^w^	11.665^x^	0.555
SEM	0.446	0.929	—

^a and b^Distinct letters within a row indicate significant differences (*P* < 0.05). ^v,w,x,y, and z^Distinct letters within a column indicate significant differences (*P* < 0.05). ^1^CHO: natural cholesterol; COPs: cholesterol oxidation products.

**Table 3:** Effect of dietary cholesterol and cholesterol oxidation products on TBARS in liver.

	TBARS in liver (MA^2^ mg/kg)		
Dietary			
CHO^1^ + COPs g/kg diet	6 weeks	12 weeks	SEM
0 g	0.564^y^	0.598^y^	0.024
1 g	0.691^x^	0.712^x^	0.016
2 g	0.736^bx^	0.832^aw^	0.021
0.9 chol. + 0.1 COPs	0.745^x^	0.740^x^	0.034
0.8 chol. + 0.2 COPs	0.695^bx^	0.833^aw^	0.032
0.5 chol. + 0.5 COPs	0.753^bx^	0.858^aw^	0.032
1.6 chol.+ 0.4 COPs	0.864^w^	0.874^w^	0.021
1.2 chol. + 0.8 COPs	0.968^v^	1.029^v^	0.020
SEM	0.032	0.016	—

^a and b^Distinct letters within a row indicate significant differences (*P* < 0.05). ^v,w,x, and y^Distinct letters within a column indicate significant differences (*P* < 0.05). ^1^CHO: natural cholesterol; COPs: cholesterol oxidation products; ^2^MA: malonaldehyde.

**Table 4:** Effect of dietary cholesterol and cholesterol oxidation products on cholesterol oxidation products in liver.

	COPs in liver (*μ*g/g)		
Dietary			
CHO^1^ + COPs g/kg diet	6 week	12 week	SEM
0 g	Trace	0.048^z^	0.003
1 g	0.030^by^	0.068^ay^	0.005
2 g	0.053^bwx^	0.109^awx^	0.006
0.9 chol. + 0.1 COPs	0.042^bx^	0.074^ay^	0.004
0.8 chol. + 0.2 COPs	0.055^bw^	0.094^ax^	0.005
0.5 chol. + 0.5 COPs	0.055^bw^	0.096^awx^	0.004
1.6 chol. + 0.4 COPs	0.054^bwx^	0.112^aw^	0.006
1.2 chol. + 0.8 COPs	0.082^bv^	0.146^av^	0.006
SEM	0.004	0.006	—

^a and b^Distinct letters within a row indicate significant differences (*P* < 0.05). ^v,w,x,y, and z^Distinct letters within a column indicate significant differences (*P* < 0.05). ^1^CHO: natural cholesterol; COPs: cholesterol oxidation products.

**Table 5:** Effect of dietary cholesterol and cholesterol oxidation products on fatty acid composition in liver.

Fatty acid (%)	0	1 g CHO	2 g CHO	0.9 g CHO + 0.1 g COPs	0.8 g CHO + 0.2 g COPs	0.5 g CHO + 0.5 g COPs	1.6 g CHO + 0.4 g COPs	1.2 g CHO + 0.8 g COPs	SEM
Myristic acid	0^d^	0^d^	0.40^c^	0^d^	0^d^	0^d^	0.63^a^	0.47^b^	0.01
Palmitoleic acid	1.10^e^	1.95^d^	2.30^bc^	2.35^bc^	2.40^bc^	2.05^cd^	2.93^a^	2.66^ab^	0.1
Palmitic acid	20.46^c^	22.39^a^	20.82^c^	21.37^b^	21.81^b^	21.42^b^	19.27^d^	19.32^d^	0.18
Linoleic acid	29.42^c^	27.95^d^	31.41^b^	26.85^e^	27.96^d^	29.42^c^	31.11^b^	32.32^a^	0.31
Oleic acid	16.66^c^	20.03^b^	18.62^b^	21.5^a^	19.99^b^	18.95^b^	19.56^b^	18.99^b^	0.36
Linolenic acid	2.29^b^	3.34^ab^	3.57^a^	3.44^ab^	3.57^a^	3.78^a^	3.45^ab^	3.58^a^	0.13
Stearic acid	22.92^a^	18.18^b^	15.89^c^	18.67^b^	17.80^b^	17.84^b^	15.69^c^	15.56^c^	0.29
Arachidonic acid	6.27^a^	5.03^c^	5.63^b^	4.99^c^	5.17^c^	5.11^c^	5.54^b^	5.58^b^	0.12
Unidentified	0.90^b^	0.94^ab^	0.96^ab^	0.84^bc^	0.92^ab^	1.03^a^	0.79^c^	0.89^bc^	0.12
SFA/USFA^2^	43.38/55.74	40.57/58.30	37.11/61.53	40.04/59.13	39.61/59.09	39.26/59.31	35.59/62.59	35.26/63.13	—

^a,b,c,d, and e^Distinct letters within a row indicate significant differences (*P* < 0.05). ^1^CHO: natural cholesterol; COPs: cholesterol oxidation products. ^2^SFA: saturated fatty acids; USFA: unsaturated fatty acids.

**Table 6:** Nutrient content of basal diet rabbit chow.

Nutrient	Content (%)
Crude protein (min.)	16.0
Crude fat (min.)	1.5
Crude fiber (min.)	17.0
Calcium (min.–max.)	20.0
Phosphorous (min.)	0.6–1.1
Salt (NaCl) (min.–max.)	0.5–1.0
Vitamin A (min.)	4,400 IU/kg

Ingredients: processed grain byproducts, forage products, roughage products, plant protein products, grain products, molasses products, calcium carbonate, salt, ferrous oxide, DL-methionine, choline chloride, vitamin E supplement, calcium pantothenate, vitamin B-12 supplement, niacin supplement, vitamin A supplement, manganese sulfate, vitamin D-3 supplement, ferrous sulfate, cobalt carbonate, calcium iodate, copper sulfate, zinc sulfate, magnesium oxide, and sodium selenite.
